# Exploring the Impact of Resistance Training at Moderate Altitude on Metabolic Cytokines in Humans: Implications for Adipose Tissue Dynamics

**DOI:** 10.3390/ijms252111418

**Published:** 2024-10-24

**Authors:** Sergio Pérez-Regalado, Josefa Leon, Paulino Padial, Cristina Benavente, Filipa Almeida, Juan Bonitch-Góngora, Blanca de la Fuente, Belén Feriche

**Affiliations:** 1 Department of Physical Education and Sport, Faculty of Sport Sciences, University of Granada, 18011 Granada, Spain; serperel284@gmail.com (S.P.-R.); ppadial@ugr.es (P.P.); cbenavente@ugr.es (C.B.); filipaalmeida@ugr.es (F.A.); juanbonitch@ugr.es (J.B.-G.); mbelen@ugr.es (B.F.); 2 Biosanitary Research Institute of Granada, ibs. Granada, 18012 Granada, Spain; 3 Clinical Management Unit of Digestive System, San Cecilio University Clinical Hospital, 18007 Granada, Spain; 4 High-Performance Centre of Sierra Nevada, Spanish Sports Council, 18196 Granada, Spain; blanca.delafuente@csd.gov.es

**Keywords:** altitude, resistance training, metabolism, cytokines, adipose tissue

## Abstract

Hypobaric hypoxia (HH) limits oxygen supply to tissues and increases metabolic demands, especially during exercise. We studied the influence of HH exposure on the subcutaneous adipose tissue (SAT) thickness and circulating metabolic-related cytokines levels after a resistance training (R_T_) program. Twenty trained men participated in a traditional hypertrophy R_T_ for 8 weeks (three sessions/week) under intermittent terrestrial HH (2320 m) or normoxia (N, 690 m) conditions. Before, at week 6, and after the R_T_, SAT, and vastus lateralis (VL) muscle thickness were measured by ultrasound. Blood samples were taken to analyse serum cytokines (IL-6, IL-15, irisin, and myostatin) by multiplex immunoassay. Our findings revealed a moderate reduction in IL-6 and irisin in HH following the R_T_ (ES < −0.64; *p* < 0.05). Additionally, R_T_ in HH promoted serum IL-15 release (ES = 0.890; *p* = 0.062), which exhibited a trivial inverse association with the reductions observed on SAT (−17.69%; *p* < 0.001) compared with N. R_T_ in HH explained ~50% of SAT variance (*p* < 0.001). These results highlight the benefit of stressor factors linked to R_T_ in HH on SAT through the modulation of serum metabolic cytokine profiles, suggesting a potential effect on overall body composition.

## 1. Introduction

Resistance exercise exerts an effective stimulus for enhancing overall metabolic health by improving glucose tolerance, insulin sensitivity, and reducing adipose tissue accumulation. Body fat accumulation could negatively impact skeletal muscle mass development, sports performance [1], and disrupt daily living tasks [2,3]. Recent investigations into the mechanisms of adipose tissue dynamics have led to the exploration of innovative strategies that integrate environmental factors to optimize exercise interventions. Among these factors, terrestrial hypobaric hypoxia (HH) presents as a unique physiological challenge that potentially influences the dynamics of subcutaneous adipose tissue (SAT) [4].

At sea level, a progressive resistance training (R_T_) program accompanied by a calorie deficit is associated with weight loss while preserving muscle mass [5]. This shift in body composition is a marker of metabolic health based on its inverse association with cardiovascular and insulin-related diseases and mortality [6]. Terrestrial hypoxia represents a potent environmental stressor that challenges human physiology in several ways [7]. At altitude, physical activity interventions resulted in significantly greater body weight loss than passive exposure or interventions at sea level [8]. This effect can largely be attributed to the enhanced energy demands at higher altitudes, where meeting nutritional intake requirements becomes increasingly difficult [9]. The influence of acute HH on lipid metabolism and inflammation is widely accepted by disrupting redox homeostasis [10], modulating blood glucose and hepatic glycogen levels, while upregulating lipid metabolism signalling pathways, such as fatty acid oxidation [11]. The resulting lipid metabolites play a key role enhancing the secretion of pro- and anti-inflammatory cytokines [12], as observed in a study carried out by our laboratory with a similar exercise program [13]. In this sense, combining R_T_ programs with the physiological implications of HH exposure could potentiate beneficial effects, especially on SAT modulation. Acute hypoxia exposure is characterized by increased lipolysis activity caused by stimulation of the sympathetic nervous system and increased glucose metabolism in human adipocytes [14]. This effect can be potentiated by adding exercise. However, the mechanism underlying the modulation of SAT by intermittent HH exposure, particularly in conjunction with R_T_ programs, remains unclear [15,16].

The low availability of oxygen in HH environments triggers multiple adaptative responses that shift towards anaerobic metabolism in adipose tissues throughout the activation of hypoxia-inducible factor 1α (HIF-1α) [17]. This transition involves the activation of systemic inflammatory and immune responses and metabolic reprogramming to maintain cellular energy homeostasis [18,19]. Furthermore, the combination of exercise under HH conditions upregulated the angio-adaptive HIF-1α response, resulting in several cytokines (IL-6, IL-15, irisin, and myostatin) release into the bloodstream [20]. During a moderate-intensity exercise stimulus, IL-6 and IL-15 exhibit analogous functions in adipose tissue, including promoting lipolysis and inhibiting preadipocyte differentiation. Conversely, both cytokines facilitate the uptake and utilization of energetic substrates in skeletal muscle [21,22]. In turn, these functions are supported by another cytokine, irisin, which facilitates energy expenditure and enhances glucose and lipid metabolism, playing a key role in the white adipose tissue browning [23]. Additionally, several authors have reported the irisin-dependent downregulation of myostatin, a negative regulator of skeletal muscle hypertrophy [24,25].

Surprisingly, despite the protective effects of R_T_ programs in maintaining muscle mass during weight loss, most previous research has studied the effects of endurance training. Moreover, to the best of our knowledge, no previous studies described the potential additive influence of HH in combination with R_T_ programs on the SAT response. Therefore, the present study aimed to investigate the effects of an 8-week R_T_ program on SAT and on the systemic response of related metabolic cytokines (such as circulating IL-6, IL-15, irisin, and myostatin) in moderate HH and normoxia (N). We hypothesized that HH would upregulate the secretion of cytokines, resulting in a more pronounced effect on SAT than the same training in N.

## 2. Results

**Figure 1:**
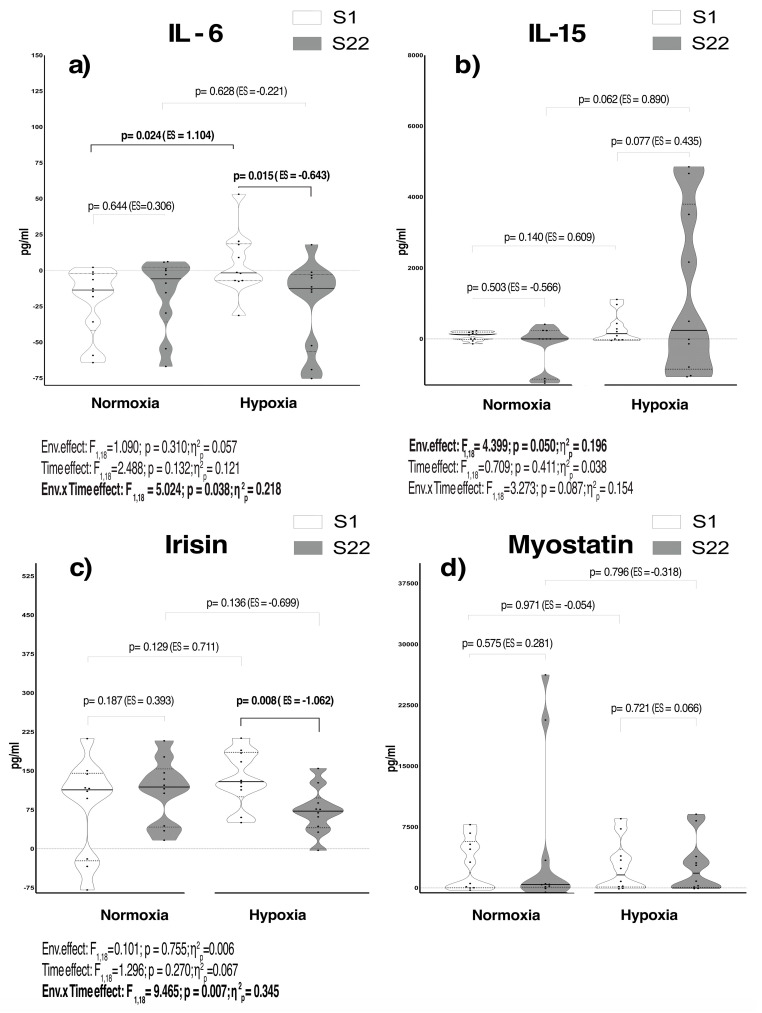
Violin plots for analysis of circulating IL-6 (**a**), IL-15 (**b**), irisin (**c**), and myostatin (**d**) levels represented as the Δ (delta scores of the peak values during recovery—baseline) before (S1) and after (S22) the R_T_ program. The black lines represent the respective group mean; error bars depict 95% CI of the mean. Dots represent single-subject data. *p*-value (*p* < 0.05), partial eta squared from ANOVA (η^2^_p_), Cohen’s d effect size (ES). ES was calculated as Δ of S22 vs. S1 or HH-N divided by the pooled standard deviation. S1: Delta change calculated as the difference between post-exercise and pre-exercise for the first training session; S22: Delta change calculated as the difference between post-exercise and pre-exercise for the last training session.

The comparison of cytokine change scores between the conditions following an 8-week R_T_ program is presented in Figure 1a–d. After the program, serum IL-6 levels displayed a large environmental x time effect (F_1,18_ = 5.024; *p* = 0.038; η^2^_p_ = 0.218). Compared with N, the pairwise comparison test yielded a large increase in IL-6 in HH at the first session (S1) (ES = 1.104; *p* = 0.024). Nevertheless, only in HH, a moderate reduction after the R_T_ program was found compared with baseline (ES = −0.643; *p* = 0.015) (Figure 1a). Circulating IL-15 revealed a large environmental effect following the R_T_ (F_1,18_ = 4.399; *p* = 0.050; η^2^_p_ = 0.196) (Figure 1b). Pairwise comparisons exhibited a clear tendency to increase circulating IL-15 in HH at the last session (S22) compared to the N condition (ES = 0.890; *p* = 0.062). A large environmental x time effect was found in irisin along the R_T_ program (F_1,18_ = 9.465; *p* = 0.007; η^2^_p_ = 0.345). Pairwise comparisons revealed a large irisin decrease in HH at S22 (ES = −1.062; *p* = 0.008) with respect to S1 (Figure 1c). Serum myostatin levels showed large individual variability, which made it difficult to detect any changes of interest (Figure 1d).

**Figure 2:**
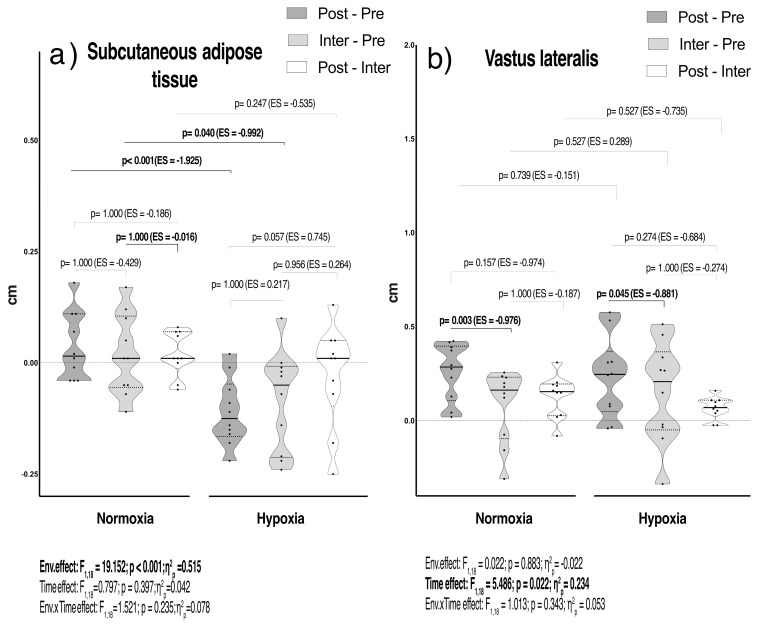
Violin plots for analysis of subcutaneous adipose tissue (**a**) and vastus lateralis (**b**) represented as the Δ of resting measurements before (Pre), at weeks 6 (Inter) and 8 (Post) of the R_T_ program. The black line represents the respective group mean; error bars depict 95% CI of the mean. Dots represent single-subject data. *p*-value (*p* < 0.05), partial eta squared from ANOVA (η^2^_p_), Cohen’s d effect size (ES). ES was calculated as (Δ of resting Post-Pre, Inter-Pre, and Post-Inter) or (HH-N) divided by the pooled standard deviation. Pre: 72 hours before R_T_ the first training session of the R_T_; Inter: week 6; Post: 72 h after the last training session of the R_T_.

Figure 2a,b displayed the R_T_ program effects on SAT and VL muscle thickness between conditions. SAT only exhibited an environmental effect (F_1,18_ = 19.152; *p* < 0.001; η^2^_p_ = 0.515). The pairwise comparison test yielded a large to very large decrease on SAT, favouring HH at Post and Inter measurements compared to N (ES < −0.99; all *p* < 0.05) (Figure 2a). Moreover, VL thickness revealed a time effect (F_1,18_ = 5.486; *p* = 0.022; η^2^_p_ = 0.234). The pairwise comparison test showed a similar increase in VL thickness in both conditions throughout the R_T_ program.

Table 1 shows the cytokine areas under the curve (AUCs) change scores throughout the 30-min recovery period after the R_T_ program. The *t*-test yielded a large decrease in IL-6 favouring HH in both the net AUC (nAUC) and incremental AUC (iAUC) after the R_T_ (ES < −0.82; all *p* < 0.05). IL-15 only displayed a very large increase in favour of HH in total AUC (tAUC) compared with N (ES = 1.17; *p* = 0.018). Although irisin and myostatin AUCs exhibited no significant differences between conditions (*p* > 0.05), a tendency to decrease was observed in irisin tAUC, favouring HH compared with N at the end of the program (ES = −0.92; *p* = 0.055).

Table 2 shows the resting AUCs change scores of VL thickness and SAT during the 8 weeks of R_T_. In HH, SAT displayed a very large in nAUC and a large decrease in iAUC, respectively, compared with N (ES < −1.01; all *p* < 0.05). VL thickness tAUC revealed a moderate but nonsignificant increase in favour of HH condition after the R_T_ program (ES = 0.79; *p* = 0.096).

Multiple linear regression models predicting SAT revealed that the altitude condition and circulating levels of irisin at S22 contributed to explaining ~60% of its variance (Table 3).

## 3. Discussion

The present study aimed to establish the potential implications of HH during the implementation of an 8-week R_T_ on SAT and VL thickness and circulating metabolic cytokine levels. The results demonstrated a more pronounced reduction on SAT when the training program was performed under HH conditions. Compared to N, large increments in circulating IL-15 were found following the training period in HH, with no clear impact on myostatin levels. In HH conditions, a progressive reduction of serum IL-6 and irisin levels throughout the training program was observed, reaching values close to baseline. These findings suggest that a R_T_ program performed under HH has a positive effect on both the reduction of SAT and the capacity to induce a favourable metabolic cytokine profile in healthy individuals.

Exposure to altitude represents a major challenge for human physiology, demanding several adaptative responses to maintain cellular homeostasis, including increased tissue oxygen delivery, ventilation, and red blood cell production [26]. Such a stressful environment is characterized by a rise in reactive oxygen species (ROS), sympathetic response, and upregulation of cytokine secretion processes [27]. Compared with N, our results revealed an increase in IL-6 secretion, which consequently returned to baseline level following the R_T_ program (Figure 1a; Table 1). It is widely accepted that short-term physiological responses derived from the resistance exercise resulted in a favourable impact on circulating IL-6 levels [28,29]. Our study found that this effect was intensified when combined with HH, thereby promoting gluconeogenesis and lipolysis processes to ensure cell survival and continuous energy supply [30]. Conversely, after 8 weeks of R_T_, a decline in IL-6 was only observed in HH. To the best of our knowledge, no previous research has described the systemic effects of a R_T_ program under intermittent HH conditions. Nonetheless, another investigation involving elite swimmers revealed a similar attenuation of serum IL-6 levels following 28 days of endurance training at moderate chronic HH exposure [31]. An association between regular training and the capacity to reduce circulating IL-6 levels has been attributed to enhanced oxidative fat metabolism and glycogen content [32,33]. As a skeletal muscle stress sensor, the gradual reduction in serum IL-6 after the R_T_ program in HH could also indicate a reduction in systemic inflammation, in addition to an increase in fat-derived energy utilization [34]. These effects have previously been reported in N conditions following chronic training, contributing to the reduction of adipose tissue IL-6 and thereby positively impacting metabolic health (i.e., an increase in glucose uptake and lipid oxidation while reducing insulin resistance) [35,36].

Contrary to N, a similar reduction trend was seen for irisin levels in HH (Figure 1c). Currently, no clear impact of R_T_ strategies on serum irisin levels has been described, making the results controversial in young populations [37]. However, it seems that elevated circulating irisin prevent muscle loss in elderly human participants after R_T_ programs [38]. Irisin is implicated in regulating energy expenditure, and its secretion depends mainly on SAT thickness [39], being sensible to fat mass, weight loss, and/or plasma glucose concentrations [40,41]. Consequently, despite similar increments in circulating irisin in both environmental conditions at the initial stages of the R_T_, a marked decline happened after the program only in HH conditions, in agreement with the reduction on SAT. It is well established that IL-15 and irisin share multiple key roles in regulating glucose/lipid homeostasis [24]. Moreover, the literature has described a positive association between elevated serum IL-15 levels and the inhibition of lipogenesis while promoting lipolysis, fat mass loss, and adipocyte size reduction [21,42] after exercise regimens in N conditions. Since muscle-derived IL-15 is highly expressed in skeletal muscle, muscle contraction derived from resistance exercise and oxidative stress [43,44,45] from moderate altitude exposure is expected to exacerbate the production of this cytokine. Accordingly, our results displayed a tendency to increase serum IL-15 following the R_T_ program in HH with respect to the N condition (Figure 1b). Moreover, total AUC revealed a large effect on circulating IL-15 favouring HH throughout the R_T_ program (Table 1). In this sense, adding the stress induced by intermittent HH exposure to R_T_ regimens appears to facilitate a SAT reduction compared to N, while promoting VL thickness development. Moreover, the secretion processes of IL-15 may exhibit greater sensitivity to the same exercise stimulus in HH compared to N, even after long-term exercise protocols, potentially attributable to an overall increase in lean mass. 

All these adaptative responses led to a more marked reduction on SAT at HH, whereas a similar increase was observed in VL thickness after R_T_ in both conditions (Supplemental Figure S1, Table 2). Furthermore, the HH condition predicted a marked reduction on SAT, explaining ~50% of its variance (Table 3, Supplemental Figure S1). Our results are consistent with previous research [46,47,48], indicating that low oxygen environments can effectively alter SAT behaviour, which is inversely correlated with myostatin expression [49]. Previous studies have shown that IL-5, IL-6, and irisin collectively contribute to reducing muscle expression and serum myostatin secretion after both acute and chronic exercise [13,24]. Moreover, it is well known that exercising at altitude elicits an increase in physiological stress, potentially involving, among other mechanisms, the upregulation of several lipid metabolic pathways driven by HIF-1α [20]. Therefore, we can suggest that the added stressor factors derived from HH exposure could alter the ratio of lipid synthesis, prioritizing fatty acid intake and oxidation [11] processes to restore energy supplies. In this sense, we can speculate that modulation of SAT induced by intermittent HH exposure combined with R_T_ programs could serve as a potential key factor in the upregulation of adipose tissue remodelling processes and in the enhancement of overall body composition.

This research has some limitations that should be noted: (1) to the best of our knowledge, no studies have investigated the combined effects of HH and long-term R_T_ protocols on metabolism-related biomarkers, limiting the ability to compare our findings with previous research; (2) the sample size was relatively small, which may have affected the range of probability distributions for the outcomes. Logistical limitations and the cost of biomarker analyses restricted the recruitment of a larger cohort. However, the findings from this specific population remain of particular interest. (3) Blood samples were collected exclusively within the 5–30-minute post-exercise window, which limits our ability to draw conclusions beyond this acute time frame; (4) SAT analyses were confined to the VL region, limiting the extrapolation of the findings to other regions.

## 4. Materials and Methods

### 4.1. Design

A longitudinal design with two independent controlled groups and intra-/inter-group measurements was used to analyse the chronic effect of a R_T_ program for 8 weeks on cytokines involved in muscle metabolism, lipid oxidation, and the regulation of adipose tissue. Participants were allocated by convenience to the N (n = 10; 690 m asl; age 22.7 ± 3.37 years; body mass 72.00 ± 7.70 kg; height 175.3 ± 4.11 cm; BMI 23.43 ± 2.51 kg/m^2^) or HH group (n = 10; 2320 m asl; age 22.8 ± 4.24 years; body mass 74.03 ± 13.87 kg; height 177.3 ± 7.40 cm; BMI 24.09 ± 4.51 kg/m^2^) [13]. 

### 4.2. Participants

Twenty physically trained sports science students familiarized with the R_T_ exercises were recruited to participate in the study. All participants had performed R_T_ a minimum of three times per week for at least the previous year. The participants had no health or muscular disorders, reported not consuming any agents associated with muscle size development during the previous month, and were unacclimated to high altitude (no altitude exposure in the 2 months before the study). All participants lived in N and were exclusively exposed to altitude during the training sessions. This study was approved by the local Ethics Committee (PEIBA: 2212-N-21) and was conducted following the Declaration of Helsinki and Biomedical Research (14/2007). Informed written consent was obtained from all participants before the beginning of the study.

### 4.3. Procedure

One week before the start of the R_T_ program, participants engaged in a preparatory session to determine the training load (70% of one repetition maximum [1RM]) for each exercise (see for further detail [13]). Two days before the beginning of the study, participants visited the laboratory for baseline anthropometric measurements (height [Seca 202, Seca Ltd., Hamburg, Germany] and body mass [Tanita TBF-300, Tokyo, Japan]) after fasting since midnight of the previous day. Testing sessions were conducted at the same time of day at a temperature of ~22 °C and ~60% of relative humidity for the N condition or ~22 °C and ~28% of relative humidity for the HH condition. Arterial oxygen saturation (SpO_2_; Wristox 3100; Nonin, Plymouth, MN, USA) was assessed per duplicate before the start of the warm-up of every R_T_ session to test the HH condition (N: 97.64 ± 1.23%, HH: 93.71 ± 1.59%; *p* < 0.001).

### 4.4. Training Program

The participants performed an 8-week R_T_ program on non-consecutive days (3 days per week; 22 sessions in total). The same R_T_ program was used for both environmental conditions. The R_T_ sessions consisted of 3 sets of 8–10 repetitions, 90 s rest between sets at 70% 1RM of six functional exercises involving the main muscle groups of the body (full-body routine: back squat, deadlift, seated cable row, wide grip pulldown, bench press, and barbell military press). A standardized warm-up of 15 min was completed at the beginning of the training session. All participants successfully completed the entire number of training sessions.

### 4.5. Ultrasound Imaging Procedure

Ultrasound measurements were obtained using an ultrasound unit (Logiq E General Electric, Chicago, IL, USA) with a linear probe 8–12 Hz before, at week 6, and after the R_T_ program. The subjects were asked not to perform strenuous exercise 72 h before the measurement day. Measurements of subcutaneous adipose tissue (SAT) and vastus lateralis (VL) muscle thickness were obtained from the subject’s dominant leg while they rested in the supine position during the entire examination.

Measurements were performed by the same investigator (CV < 1.8%), who was blinded to all time points. The ultrasound transducer was placed longitudinally at 50% between the greater trochanter of the femur and the lateral epicondyle of the knee on the VL [50]. Three images were taken and stored in each time point measurement. An indelible marker was used to denote relevant landmarks on the leg to ensure that the probe was placed on the same muscle region at each visit. The same ultrasound depth was maintained for all measurements for each participant, ranging from 0 to 8 cm. The mean of the three measurements was used for statistical analysis.

The images were analysed using the imaging analyses software Fiji (Fiji ImageJ2 version: 2.9.0/1.53t, Bethesda, MD, USA) [51,52]. ImageJ scale was determined based on the depth of the ultrasound image. Muscle thickness was measured as the distance between the bottom of the superficial aponeurosis of the VT and the top of the deep aponeurosis. The intraclass correlation coefficient for interrater reliability was greater than 0.95 for VL muscle thickness. SAT was measured vertically between the cutis and superficial aponeurosis of the VL and within three locations at 25%, 50%, and 75% of the view. The measurements were collected one time and averaged together [53].

### 4.6. Blood Samples

Blood samples were collected at baseline and in minutes 5 and 30 after the first (S1) and last (S22) R_T_ session. All blood sample collections were taken after 12 h of fasting. Circulating IL-6, IL-15, irisin, and myostatin levels were determined. Peak values for each studied variable throughout the post-exercise recovery window-time were analysed. Immediately after exercise, an antecubital forearm vein was canalized via a catheter and remained permeable using a physiological saline solution. Two millilitres of blood before each extraction were discarded to avoid sample dilution. All blood samples were kept in cold conditions after extraction and centrifuged to isolate serum within 4 h at 3000 rpm for 10 min. Finally, several 500 µL aliquots of serum were stored at −70 °C until use. The pre-exercise condition was established 72 h before the beginning of the study at N conditions after 2 days of refraining from any exercise.

Circulating IL-6 concentrations were assessed using the Milliplex Human High Sensitivity T Cell Panel (HSTCMAG-28SK) from Sigma-Aldrich (Darmstadt, Germany). The detection range was 0.11–8.17 pg/mL. Serum Il-15 levels were quantified by Invitrogen™ (Wien, Austria) IL-15 Human ELISA Kit (P40933). The detection range was 15.6–1000.0 pg/mL. Circulating irisin was determined using an irisin enzyme immunoassay kit (EK-067-29; Phoenix Pharmaceuticals, Burlingame, CA, USA). The detection range was 2.73–96 pg/mL. Myostatin analyses were performed using the GDF-8/Myostatin Quantikine ELISA Kit (DGDF80) from R&D Systems (Minneapolis, MN, USA). The detection range of the Myostatin kit is 0.922–5.32 pg/mL. Quantitative data were obtained using the Luminex-200 system (Luminex Corporation, Austin, TX, USA), and data analysis was performed with Luminex 100™ IS v2.3 software. All procedures followed the manufacturer’s instructions. All analyses were performed in N using the same equipment by specialized staff.

### 4.7. Statistical Analyses

The data are presented as the mean difference ± standard deviation (SD). Data normality assumptions were tested using the Shapiro–Wilk test (*p* < 0.05). We employed the three-sigma rule of thumb method to determine the presence of outliers in the raw data [54].

Firstly, differences between the environmental conditions following the R_T_ session were interpreted through repeated measures analysis of variance (ANOVA). One-factor ANOVA was used to assess the environmental condition (HH vs. N) on the delta change (Δ) of cytokines (IL-6, IL-15, and irisin) between conditions. Delta change was calculated as the difference (post-exercise–pre-exercise) of the peak values between the last and first training sessions (ΔS22–S1). Non-normally distributed variables (myostatin) were compared similarly but using the Mann–Whitney U test (between groups) or Wilcoxon test (within groups) [55].

A two-factor ANOVA with one within-group factor (moment: with 3 levels [Δ Post-Pre vs. Δ Inter-Pre vs. Δ Post-Inter values]) and one intergroup factor (environmental condition: with 2 levels [HH vs. N]) was used to assess the effect of the program on muscle thickness and subcutaneous adipose tissue measurements (SAT and VL). Significant main effects and interactions were subsequently analysed using Bonferroni post hoc tests. When the sphericity assumption for ANOVA was violated, we applied the Greenhouse–Geisser correction. Partial eta squared for main effects (η^2^_p_) was obtained from the ANOVA and was interpreted as ≥0.01 (small), ≥0.06 (medium), and ≥0.14 (large) [56]. Significant main effects and interactions were analysed with the Bonferroni post hoc test.

Secondly, change scores for the pre- and post-R_T_ program data were determined using the total (tAUC), net (nAUC), and incremental area under the curve (iAUC) methods. Independent-sample *t*-tests were used to evaluate the within-group R_T_ program effect (Δ calculated as the recovery times periods from S22–S1 AUCs) to identify differences between the conditions (HH vs. N) for cytokines (IL-6, IL-15, irisin, and myostatin), SAT, and VL thickness.

Complementary to the previous comparison tests, the magnitude of the changes was quantified using the standardized differences based on Cohen’s effect sizes (ES). These measures were calculated as the mean change between measurements (HH-N; or post-exercise–pre-exercise absolutely peak value; or Post-Pre, Inter-Pre, and Post-Inter values) divided by the pooled SD for all pair comparisons. The thresholds for interpretation of Cohen’s d were set as follows: <0.2 (trivial), 0.21–0.5 (small), 0.5–0.8 (moderate), 0.8–1.3 (large), and >1.30 (very large) [57].

Predictions for SAT adaptations were estimated from the condition, muscle architecture, and serum variables using multiple linear regression models using a stepwise approach.

All data extraction and analyses were performed using R Statistics version 2023.06.1+524 (Boston, MA, USA) and IBM SPSS Statistics version 28.0.1.0 for MacOS (IBM Corp., Armonk, NY, USA). The level of significance was set at *p* < 0.05.

## 5. Conclusions

In summary, our findings revealed that a R_T_ period during hypoxia significantly reduced SAT thickness and altered the secretion of metabolic-related cytokine profiles with respect to the same training in N. The expected overall increase in lean mass could explain the gradual reduction in irisin release and the promotion of muscle IL-15 secretion attributed to the stress of the HH combined with R_T_. Surprisingly, VL thickness values exhibited similar increments in both conditions following the R_T_. This outcome must be considered with caution due to the specific portion of muscle studied; therefore, further consideration is required in whole-body composition evaluation to draw firm conclusions. Moreover, the absence of changes in myostatin levels was consistent with the inhibitory effect on both circulating IL-6 and irisin, which were slightly elevated at the initial stages of the intervention in HH. Thus, taking all the results together, it appears that the stressor factors linked to R_T_ in HH may play a pivotal role in modulating SAT thickness. Further research targeting this potential non-pharmacological approach for adipose regulation is needed to determine its effectiveness for combating overweight and obesity-related disorders.

## Figures and Tables

**Table 1:** Circulating metabolic cytokines (IL-6, IL-15, irisin, and myostatin) areas under the curve before and following 8 weeks of R_T_ in hypoxia and normoxia.

		Recovery AUCs Δ S22–S1		HH vs. N *p* Value (ES)
		N (Means ± SD)	HH (Means ± SD)	
IL-6 (pg/mL)	tAUC	86.36 ± 118.92	23.84 ± 113.43	0.353 (−0.54)
	nAUC	7.19 ± 28.01	−33.33 ± 63.70	**0.043 (−0.82)**
	iAUC	0.61 ± 1.61	−6.44 ± 9.13	**0.015 (−1.08)**
IL-15 (pg/mL)	tAUC	244.82 ± 1771.61	3930.38 ± 4104.48	**0.018 (1.17)**
	nAUC	12.83 ± 64.20	1896.70 ± 3358.02	0.143 (0.79)
	iAUC	−37.56 ± 127.03	1795.83 ± 2540.69	0.579 (1.02)
Irisin (pg/mL)	tAUC	74.55 ± 120.82	−75.07 ± 196.78	0.055 (−0.92)
	nAUC	59.77 ± 184.52	−66.94 ± 143.05	0.103 (−0.77)
	iAUC	29.92 ± 140.09	−74.85 ± 126.48	0.096 (−0.78)
Myostatin (pg/mL)	tAUC	12.57 ± 13116.45	−9118.47 ± 43,325.29	0.796 (−0.28)
	nAUC	−1133.58 ± 6612.84	−3074.81 ± 14,631.56	0.684 (−0.17)
	iAUC	−1140.29 ± 6577.39	−3385.80 ± 14,304.45	0.631 (−0.20)

*p*-value (*p* < 0.05); pg/mL: picograms per millilitre; SD: standard deviation; Cohen’s d effect size (ES).

**Table 2:** Ultrasound subcutaneous adipose tissue and vastus lateralis thickness areas under the curve before and following 8 weeks of R_T_ in hypoxia and normoxia.

		Basal AUCs Δ S22–S1		HH vs. N *p* Value (ES)
		N (Means ± SD)	HH (Means ± SD)	
Subcutaneous adipose tissue (cm)	tAUC	0.66 ± 0.38	0.74 ± 0.38	0.647 (0.21)
	nAUC	0.04 ± 0.14	−0.14 ± 0.14	**0.009 (−1.34)**
	iAUC	0.20 ± 0.21	0.04 ± 0.09	**0.041 (−1.01)**
Vastus lateralis muscle thickness (cm)	tAUC	5.65 ± 0.76	6.31 ± 0.91	0.096 (0.79)
	nAUC	0.21 ± 0.26	0.27 ± 0.37	0.669 (0.19)
	iAUC	0.26 ± 0.16	0.32 ± 0.30	0.605 (0.23)

*p*-value (*p* < 0.05); SD: standard deviation; cm: centimetres; Cohen’s d effect size (ES); tAUC: total area under the curve, nAUC: net area under the curve: iAUC: incremental area under the curve.

**Table 3:** Multiple linear regression models predicting subcutaneous adipose tissue.

Multiple Linear Regression Models Predicting SAT				
	Model 1		Model 2	
	Estimate (CI)	Std. Error	Estimate (CI)	Std. Error
**(Constant)**	0.184 ** (0.71, 0.297)	0.054	0.073 (−0.061, 0.208)	0.064
**Condition**	−0.147 *** (−0.219, −0.075)	0.034	−0.119 ** (−0.186, −0.052)	0.032
**Irisin S22**			0.001 * (0.000, 0.001)	0.000
**n**	20		20	
**R^2^/adj. R^2^**	0.507/0.480		0.645/0.604	
**F-statistic**	18.520 ***		6.628 **	

95% CI: confidence interval; tAUC: total area under the curve; nAUC: net area under the curve: iAUC: incremental area under the curve; * *p* < 0.05, ** *p* < 0.01, *** *p* < 0.001.

## Data Availability

The data that support the findings of this study are available from the corresponding author upon reasonable request.

## Supplementary Materials

The following supporting information can be downloaded at: https://www.mdpi.com/article/10.3390/ijms252111418/s1.
