# Supplementary material for: Exploring the Impact of Resistance Training at Moderate Altitude on Metabolic Cytokines in Humans: Implications for Adipose Tissue Dynamics
Source: Int J Mol Sci. 2024 Oct 24;25(21):11418. doi: 10.3390/ijms252111418 (PMC11546518; doi:10.3390/ijms252111418)
Supplement: Supplementary file 1 [file ijms-25-11418-s001.zip › ijms-3248825-supplementary.pdf]

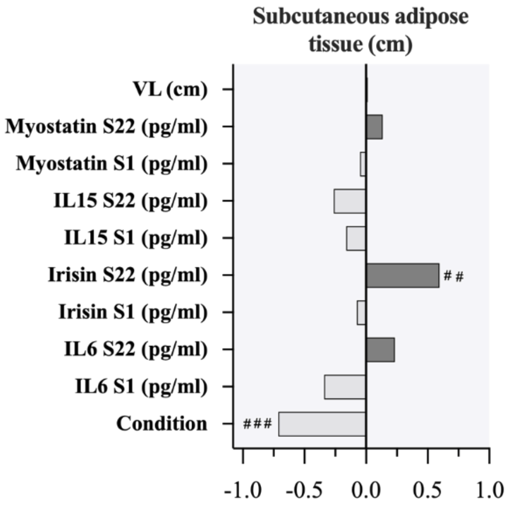

Figure S1: Pearson's correlation coefficients between SAT and circulating metabolic cytokines (IL-6, IL-15, irisin, and myostatin) and VL thickness parameters. SAT: subcutaneous adipose tissue; VL: vastus lateralis; S: session. P-value ($p<0.05$). $p < 0.01$ ##; $p < 0.001$ ###.
